# Supplementary material for: Epicardial Adiposity in Relation to Metabolic Abnormality, Circulating Adipocyte FABP, and Preserved Ejection Fraction Heart Failure
Source: Diagnostics (Basel). 2021 Feb 26;11(3):397. doi: 10.3390/diagnostics11030397 (PMC7996796; doi:10.3390/diagnostics11030397)
Supplement: Supplementary file 1 [file diagnostics-11-00397-s001.zip › diagnostics-1086263-supplementary.pdf]

Supplemental Materials

Epicardial Adiposity in Relation to Metabolic Abnormality, Circulating Adipocyte FABP, and Preserved Ejection Fraction Heart Failure

Jiun-Lu Lin, Kuo-Tzu Sung, Yau-Huei Lai, Chih-Hsuan Yen, Chun-Ho Yun, Cheng-Huang Su, Jen-Yuan Kuo, Chia-Yuan Liu, Chen-Yen Chien, Ricardo C. Cury [10], Hiram G. Bezerra and Chung-Lieh Hung

Baseline Characteristics and Echocardiographic Profiles of Different HF Risk Groups

Table S1. Characteristics of the study cohort at enrollment.

	Variables	Control (n = 40)	High-Risk (n = 161)	HFpEF (n = 51)	p (Trend)	p (ANOVA)
Anthropometric Measurements	Female sex, n (%)	32 (64.0%)	89 (60.1%)	42 (82.4%)	-	0.042
	Age, years	59.2 ± 5.8	65.7 ± 9.5 *	71.2 ± 10.5 *†	<0.001	<0.001
	Systolic blood pressure, mm Hg	124.7 ± 16.5	141.8 ± 18.4 *	147.5 ± 21.3 *	<0.001	<0.001
	Diastolic blood pressure, mm Hg	76.1 ± 11.2	81.7 ± 13.6	81.3 ± 13.6	0.11	0.16
	Heart rate, min^{-1}	72.6 ± 10.9	76.2 ± 11.4	76.7 ± 11.8	0.08	0.10
	Waist circumference, cm	78.8 ± 9.1	91.0 ± 11.4 *	93.0 ± 11.2 *	<0.001	<0.001
	Weight	57.3 ± 10.1	68.7 ± 13.4 *	66.0 ± 10.9 *	0.003	<0.001
	BMI, kg/m^2	22.3 ± 2.7	27.2 ± 4.1 *	27.4 ± 3.8 *	<0.001	<0.001
	Body fat, %	26.4 ± 6.8	35.3 ± 8.7 *	38.2 ± 9.2 *	<0.001	<0.001
Biochemistry Measurements	Fasting glucose, mg/dL	93.0 ± 8.6	110.7 ± 28.1 *	138.9 ± 59.7 *†	<0.001	<0.001
	Total cholesterol, mg/dL	213.4 ± 44.6	197.3 ± 41.8	192.0 ± 43.2	0.023	0.06
	Triglyceride, mg/dL	71.5 ± 56.8	115.9 ± 73.6 *	146.1 ± 122.9 *	<0.001	<0.001
	HDL, mg/dL	64.7 ± 18.5	54.0 ± 19.1 *	49.8 ± 18.0 *	<0.001	<0.001
	LDL, mg/dL	133.7 ± 36.9	119.0 ± 34.1	111.6 ± 37.5 *	0.004	0.012
	Uric acid, mg/dL	5.4 ± 0.9	5.8 ± 1.4	6.9 ± 1.7	<0.001	<0.001
	e-GFR, mL/min/1.73 m^2	90.4 ± 15.2	80.6 ± 25.2 *	65.6 ± 29.2 *†	<0.001	<0.001

Special Biomarkers	hs-CRP, mg/L	0.12 ± 0.14	0.21 ± 0.22	0.33 ± 0.32	<0.001	<0.001
	BNP, pg/mL	12.7 ± 15.5	32.9 ± 44.3	191.8 ± 222.1 *†	<0.001	<0.001
	Galectin-3, ng/mL	1.56 ± 1.14	2.62 ± 1.92 *	4.04 ± 3.50 *†	<0.001	<0.001
	PIIINP, ng/mL	0.69 ± 0.14	0.96 ± 0.31 *	1.28 ± 0.51 *†	<0.001	<0.001
	FABP	16.3 ± 5.7	24.4 ± 20.0	39.3 ± 26.9 *†	<0.001	<0.001
Past Histories	Hypertension, *n* (%)	0 (0%)	132 (82.0%)	47 (92.6%)	-	<0.001
	Diabetes, *n* (%)	0 (0%)	46 (28.6%)	29 (56.9%)	-	<0.001
	Cardiovascular disease, *n* (%)	0 (0%)	19 (11.8%)	15 (29.4%)	-	<0.001
Cardiac Structure	EAT thickness, mm	6.4 ± 1.9	8.2 ± 1.5 *	9.7 ± 1.65 *†	<0.001	<0.001
	IVS, mm	8.2 ± 0.8	9.2 ± 1.3 *	10.1 ± 1.8 *†	<0.001	<0.001
	LVPW, mm	8.1 ± 0.8	9.2 ± 1.3 *	9.9 ± 1.2 *†	<0.001	<0.001
	LVIDd, mm	44.4 ± 3.8	46.7 ± 3.7 *	46.2 ± 4.4 *	0.057	0.004
	LV mass, g	115.0 ± 25.4	147.0 ± 34.4 *	160.3 ± 40.1 *†	<0.001	<0.001
	LV mass index, gm/m^2	68.3 ± 16.3	79.2 ± 18.2 *	88.4 ± 18.3 *†	<0.001	<0.001
	LVH by Echo (%)	0 (0%)	14 (8.7%)	13 (25.5%)	-	<0.001
Diastolic Function	LA volume (max), mL	29.0 ± 9.1	36.2 ± 15.8 *	50.2 ± 17.9 *†	<0.001	<0.001
	E/A ratio	1.07 ± 0.42	0.87 ± 0.27 *	0.95 ± 0.49	0.20	0.01
	Tau (T)	28.4 ± 5.0	35.7 ± 9.9 *	45.0 ± 14.0 *†	<0.001	<0.001
	DT, ms	206.9 ± 39.35	205.8 ± 39.90	214.8 ± 50.54	0.35	0.43
	IVRT, ms	90.88 ± 15.04	89.76 ± 13.92	94.13 ± 13.85	0.25	0.18
	TDI-e′ (average), cm/s	9.1 ± 1.7	7.8 ± 1.8 *	6.4 ± 1.4 *†	<0.001	<0.001
	E/e′ (average)	7.1 ± 1.7	9.5 ± 2.9 *	13.2 ± 4.3 *†	<0.001	<0.001
	LV SRe	1.29 ± 0.34	1.09 ± 0.28 *	0.9 ± 0.23 *†	<0.001	<0.001
	LV SRa	1.16 ± 0.9	1.21 ± 0.23	1.16 ± 0.32	0.9	0.29
Systolic Function	TDI-s′ (average), cm/s	8.0 ± 1.36	7.79 ± 1.37	6.72 ± 1.41 *†	<0.001	<0.001
	GLS, %	−21.0 ± 2.00	−19.95 ± 2.08 *	−16.75 ± 2.50 *†	<0.001	<0.001
	GCS, %	−21.33 ± 2. 40	−20.75 ± 2.75	−19.72 ± 3.58	0.008	0.026
	LV SRs	−1.24 ± 0.13	−1.13 ± 0.14 *	−1.02 ± 0.11 *†	<0.001	<0.001
	SV, mL	62.1 ± 12.7	67.8 ± 11.6 *	65.5 ± 13.8	0.29	0.028
	LV ejection fraction (%)	77.5 ± 8.9	78.3 ± 8.0	77.1 ± 10.1	0.77	0.69

A, late diastolic filling velocity; BMI, body mass index; BNP, B-type natriuretic peptide; DT, deceleration time; e-GFR, estimated glomerular filtration rate; E, early diastolic filling velocity; EAT, epicardial adipose tissue; E/E', relationship between maximal values of passive mitral inflow (E, PW-Doppler) and lateral early diastolic mitral annular velocities (E′, TDI); FABP, fatty acid-binding protein; GCS, global circumferential strain; GLS, global longitudinal strain; HDL, high-density lipoprotein cholesterol; hs-CRP, high-sensitivity C-reactive protein; IVRT, isovolumic relaxation time; IVS, interventricular septum; LA, left atrium; LDL, low-density lipoprotein cholesterol; LV, left ventricle; LVIDd, left ventricular internal diameter end diastole; LVIDs, left ventricular internal diameter end systole; LVPW, left ventricle posterior wall; PIIINP, N-terminal pro-peptide of type III procollagen; S′, peak systolic mitral annular velocity; SR, strain rate; SVi, stroke volume index; Tau (T), time constant of LV isovolumic pressure decline. Sex and percent with hypertension, diabetes, and coronary artery disease are analyzed via chi-square test. * $p < 0.05$ at-risk group or HFpEF versus control by ANOVA with Bonferroni post hoc test, † $p < 0.05$ between at-risk group and HFpEF by ANOVA with Bonferroni post hoc test.

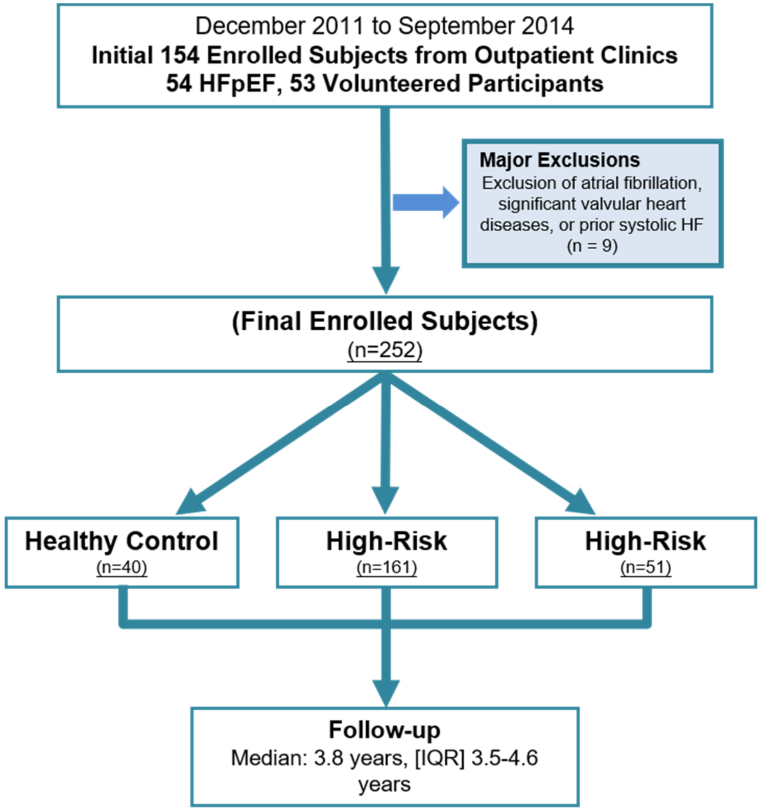

Figure S1. The flowchart of the patient enrollment process.

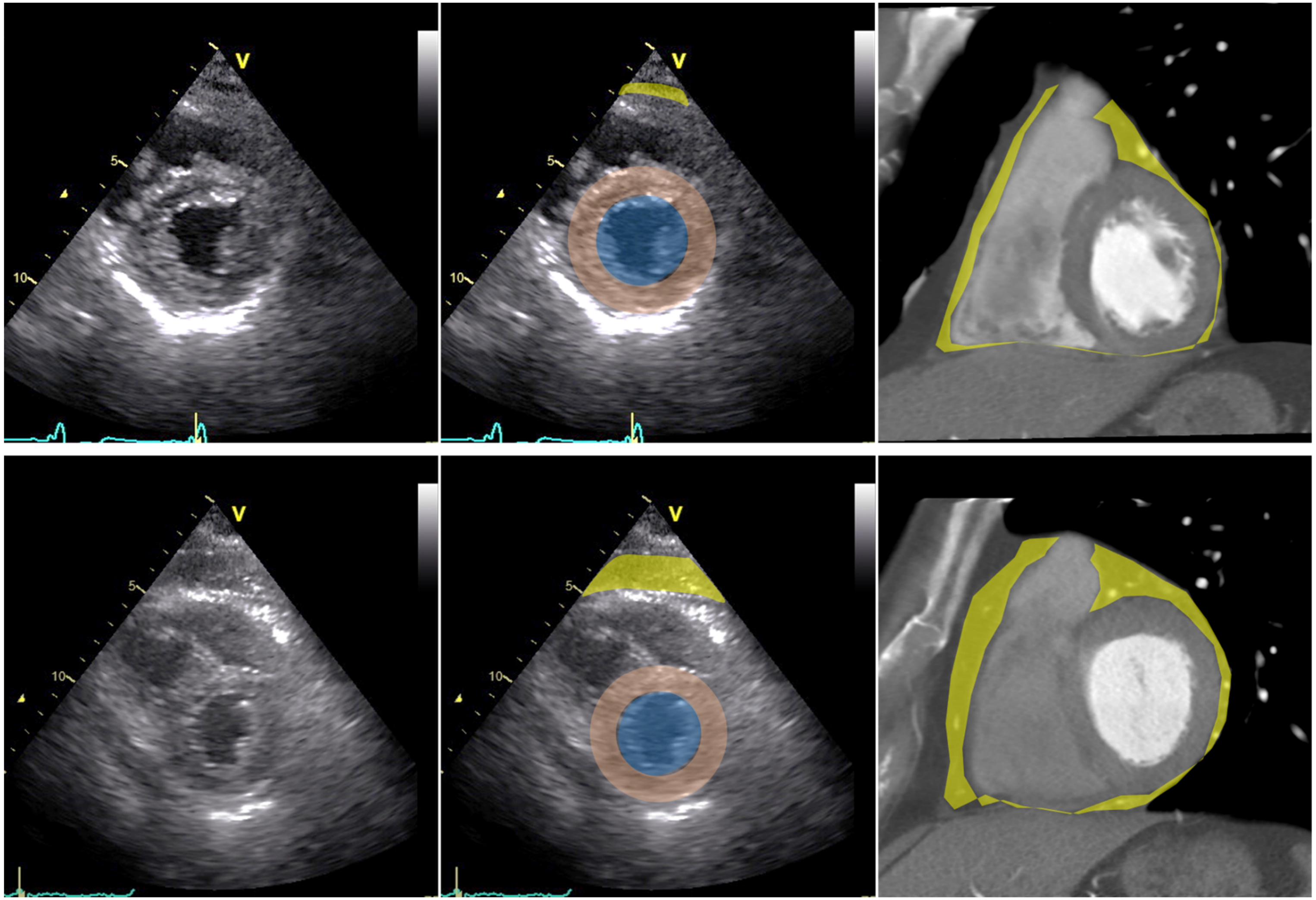

Figure S2. Schematic illustration of echocardiography-derived EAT measures (yellow regions). A representative healthy control (65-year-old healthy male patient, upper panel). A clinical example of HFpEF (78-year-old female patient, lower panel). Validating EAT with CT-based PCF measurements were shown for correspondent echo case from the 65-year-old healthy control and the 78-year-old HFpEF patient.

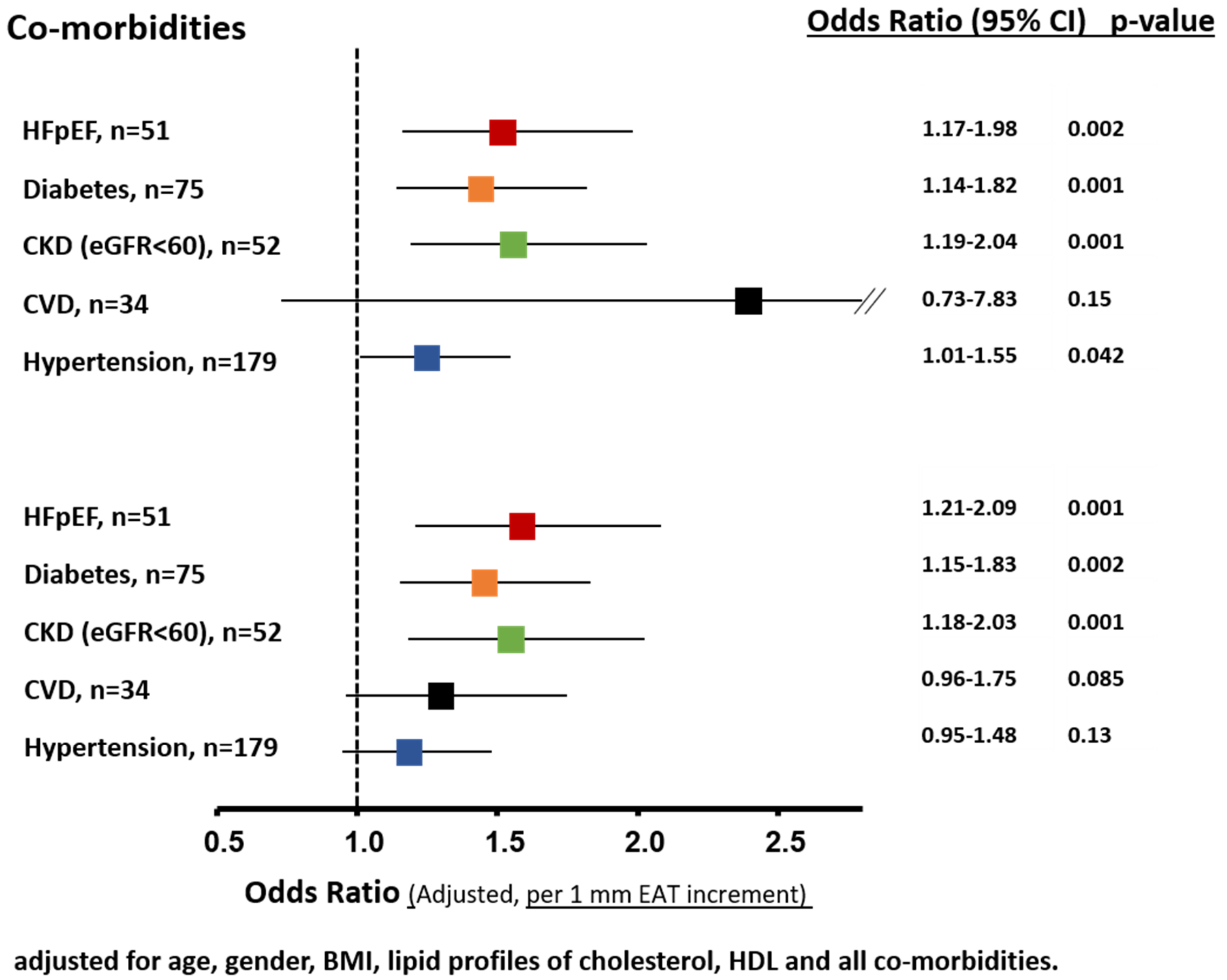

Figure S3. Adjusted odds ratios for clinical co-morbidities by per 1 millimeter EAT increment. Individuals with increased EAT measures have significantly higher odds for HFpEF, diabetes and CKD. CKD = chronic kidney disease; EAT, epicardial adipose tissue; HFpEF = heart failure with preserved ejection fraction.

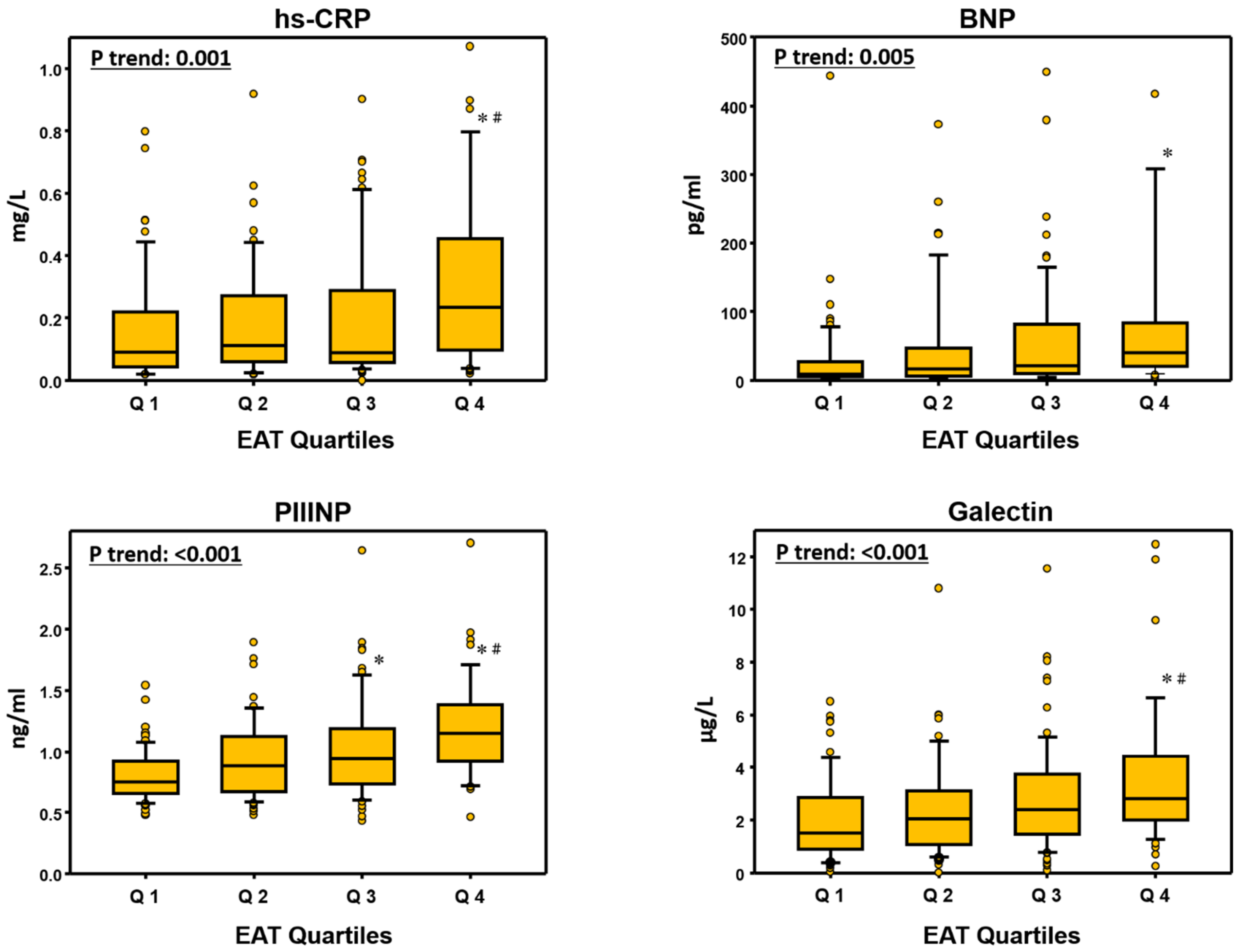

* p value<0.05 as compared with "Q1", # p value<0.05 as compared with "Q2".

Figure S4. Box-and-whisker plots of plasma hs-CRP, BNP, PIIINP and Galectin level by quartiles of EAT. BNP, B-type natriuretic peptide; EAT, epicardial adipose tissue; hs-CRP, high sensitivity C-reactive protein; PIIINP, procollagen type III N-terminal peptide . Quartiles of EAT correspond to the following values: Q1 < 7.2 mm; Q2 ≧7.2 and＜8.4 mm ; Q3 ≧8.4 and＜9.5 mm and > 27.5; Q4 ≧ 9.5 mm.
